# Culturally diverse families of young children with ASD in Sweden: Parental explanatory models

**DOI:** 10.1371/journal.pone.0236329

**Published:** 2020-07-27

**Authors:** Rano Zakirova-Engstrand, Tatja Hirvikoski, Mara Westling Allodi, Lise Roll-Pettersson

**Affiliations:** 1 Department of Special Education, Stockholm University, Stockholm, Sweden; 2 Department of Women’s and Children’s Health, Pediatric Neuropsychiatry Unit, Center for Neurodevelopmental Disorders at Karolinska Institutet (KIND), Karolinska Institutet, Stockholm, Sweden; 3 Habilitation & Health, Region Stockholm, Stockholm, Sweden; 4 Center for Psychiatry Research, Region Stockholm, Stockholm, Sweden; Radboud University, NETHERLANDS

## Abstract

**Background:**

Research suggests that families’ knowledge and cultural perceptions of autism spectrum disorder (ASD), and beliefs about its etiology and prognosis, can affect parents’ recognition of the first signs of autism in their children and influence help seeking and treatment decisions.

**Objective:**

This study investigated explanatory models of autism among parents of young children with ASD in the multicultural context of Sweden.

**Method:**

Seventeen parents from diverse cultural, ethnic and linguistic backgrounds participated in semi-structured interviews. A deductive approach to qualitative content analysis was used to analyze data. Five domains of the Explanatory Model supplementary module of the Cultural Formulation Interview (CFI) were used as coding categories, operationalized as ‘Parents’ understanding of autism’; ‘Autism prototypes’; ‘Causal explanations’; ‘Course of autism’, and ‘Help seeking and treatment expectations’

**Results:**

The results showed that parents’ prior knowledge of autism and experience of young children’s typical developmental trajectories, as well as the opinions of children’s grandparents and preschool teachers, affected symptom recognition and help seeking. There were differences in parents’ explanatory models before and after ASD diagnosis. Initial interpretations of the disorder included medical conditions and reaction to environmental influences, while genetic, supernatural/religious factors, and vaccinations were mentioned as definite causes after obtaining a clinical diagnosis. Parents also held multiple explanatory models, influenced by the views of family members and information obtained from media or from health care professionals. Parents’ treatment decisions included use of available state-funded support services, and complementary and alternative treatments.

**Conclusion:**

The results demonstrate the utility of the CFI’s Explanatory Model supplementary module in autism research. Implications for clinical practice are discussed.

## Introduction

Autism spectrum disorder (ASD) is a neurodevelopmental, lifelong condition characterized by core features in two domains: impairments in social communication and the presence of restricted, repetitive behaviors and interests across multiple contexts [1]. It is also characterized by heterogeneity, implying that individuals diagnosed with ASD can demonstrate a wide variation in behavioral representation and differences in cognitive and language developmental profiles, as well as presence or absence of coexisting conditions [2]. The onset of symptoms appears in early childhood, when ASD symptoms are present in the first 12 months of life (early onset) or when already acquired skills are lost during the first few years of life (regressive onset) [3]. While the etiology of ASD has not been fully established [4], it has been suggested that a complex interplay of genetic and environmental factors might contribute to the cause of the disorder [5]. In the absence of identified etiologies, a variety of developmental, educational, behavioral, and complementary and alternative interventions have been proposed to address ASD symptoms [6]. However, information overload on a plethora of existing interventions can be overwhelming and sometimes even misleading for parents of children with ASD [7, 8]. As Hebert and Koulouglioti [6] have noted, “Without definite information on the cause, course or treatment of autism, parents of children with autism are left to come to their own interpretation of the disorder” (p. 150). This in turn can lead parents to choose treatments that might lack sufficient evidence of efficacy or safety [9].

Research suggests that cultural background can be an important variable impacting parents’ beliefs about their child’s condition, their recognition and interpretation of ASD symptoms, as well as their beliefs about the etiology and prognosis of autism, which can in turn affect their treatment decisions [3, 9, 10]. It has also been suggested that families’ cultural characteristics can influence their access to various educational and health care services within a certain cultural context [11, 12]. However, research on the impact of culture on parental beliefs about the cause and course of autism, and the relationship between these beliefs and families’ decisions regarding choice of interventions for their children diagnosed with ASD, is still limited [6], both internationally and in the context of Northern European countries. The present study addresses this gap, and reports findings on the views of a culturally diverse group of parents of young children with ASD in Sweden about the reasons for their child’s autism, their understanding of the course of autism, help-seeking behaviors, and treatment choices. To situate our findings, in the background section we first describe the theoretical and conceptual frameworks that guided our investigation; we then provide a brief overview of support services available to children with ASD and their families in Sweden.

### Explanatory models of illness/chronic condition

Informed by anthropological and cross-cultural studies of perceptions of chronic medical conditions, Kleinman [13] proposed a theoretical framework known as explanatory models of illness. Kleinman [13] defined explanatory models as “the notions about an episode of sickness and its treatment that are employed by all those engaged in the clinical process” (p. 105). He emphasized five major aspects of explanatory models of illness or chronic condition: (1) etiology; (2) time and mode of onset of symptoms; (3) pathophysiology; (4) course of illness (including type and degree of severity); and (5) treatment. Kleinman [13] argued that cultural factors strongly influence people’s perceptions and explanations of illness or chronic condition, as well as coping strategies based on those explanations. Explanatory models reflect cultural and religious beliefs, social class, educational and professional background, and past experiences with illness and interaction with health care systems. Kleinman noted that a patient’s explanatory models may be inconsistent and self-contradictory; moreover, one and the same patient can have multiple explanatory models for his/her illness. Kleinman et al. [14] maintained that explanatory models of illness may differ between professionals and patients or their family members, which can become either a barrier to or a facilitator of joint approaches to health care. In ideal situations, patients’ and professionals’ explanatory models should concur. Professionals’ lack of understanding of or inattention to the patient’s experiences of illness or disability may lead to non-compliance and dissatisfaction with care [15]. To elicit patients’ explanatory models, health care professionals are recommended to ask a set of specific questions, e.g. *What do you think has caused your problem*? *Why do you think it started when it did*? *Do you think it will have a short or long course*? *What kind of treatment do you think you should receive*? (S1 Table). Further extensive ethnographic fieldwork expanded Kleinman’s initial framework of explanatory models and laid the ground for the development of several cultural assessment instruments [16], e.g. the Explanatory Model Interview Catalogue (EMIC) and the McGill Illness Narrative Interview (MINI). The latter instrument [17] reflects a broadened view on the types of knowledge that may contribute to the formation of patients’ explanatory models. As Kirmayer et al. [18] argue, patients (and their family members) may explain symptoms using not only causal logic; they may also reason analogically through images and metaphors based on prototypical experiences of self or others (e.g. family, social or media prototypes). The concept of illness prototypes is also reflected in the most recent approach to cultural assessment based on Kleinman’s work: the Cultural Formulation Interview (CFI), an essential part of the latest edition of the Diagnostic and Statistical Manual of Mental Disorders (DSM-5) [1, 19], where ASD is one of the included conditions.

#### Explanatory models in the DSM-5

The DSM-5, published in 2013, introduced an approach to cultural assessment–the Cultural Formulation Interview (CFI)–that expands the Outline for Cultural Formulation (OCF) included in the previous edition of the Manual, the DSM-IV [20]. The CFI is an evidence-based tool that aims to assist clinicians in making person-centered cultural assessments to inform diagnosis and treatment planning by systematically collecting information on the cultural identity of the individual; cultural conceptualization of distress; psychosocial stressors and cultural features of vulnerability and resilience; and cultural features of the relationship between the individual and the clinician [1]. The CFI consists of three types of semi-structured interviews: (1) the core interview protocol of 16 open-ended questions; (2) the ICF-Informant Version to obtain information from caregivers, parents (if the individual is a young child), relatives, and/or friends; and (3) 12 supplementary modules to obtain more information to complement basic assessment [21]. Both the core CFI and its informant version ask the patient (or members of his/her social network) to define his/her health problem in order to obtain a multitude of explanations, including nonmedical descriptions given by the patient and people in his/her closest environment [22]. The interview protocols also ask questions to identify any cultural factors that may have affected self-coping and help seeking in the past, including medical care, mental health care, support groups, religious or spiritual groups, traditional healers, and others. Questions are asked to identify cultural factors that may affect the patient’s current help seeking, ideas about current treatment preferences, and relationships with clinicians. The CFI’s first supplementary module–the Explanatory Model–expands the core CFI with the aim to help clinicians to conduct a more comprehensive cultural assessment of the patient’s own ideas about the causes of their health problem, the perceived effects of the problem over time, and their views on the most efficacious treatments [19]. Hinton et al. [19] emphasize that the use of the module can help “avoid the error of decontextualization–the mistake of taking the presented problem out of its culturally shaped social and meaning context” (p.65). The module consists of 14 questions structured around five domains: (1) General understanding of the problem (the person’s description of his/her condition, as well as descriptions of beliefs about the person’s condition held by people in his/her immediate social environment); (2) Illness prototypes; (3) Causal explanations; (4) Course of illness (the person’s expectations about the course of his/her condition—acute, chronic, or impaired [13]); and (5) Help seeking and treatment expectations (S2 Table). The use of both the core CFI and the Explanatory Model supplementary module can be clinically useful in several aspects, e.g. in maximizing treatment adherence, building empathy, and addressing stigmatization [19].

In the field of autism, several authors have recommended using Kleinman’s [13] explanatory model framework to understand parents’ perceptions of their child’s autism, causal explanations, beliefs about prognosis, and treatment preferences. For instance, in clinical practice, Levi et al. [23] suggest using the set of eight questions proposed by Kleinman [13] in its modified form to inform diagnostic assessment and treatment planning for ASD (S3 Table). These questions can guide clinicians’ interactions with families from diverse cultural backgrounds and aid understanding of parental beliefs about ASD, which might help to tailor appropriate interventions for children and their families. Mandell and Novak [9] caution that if parents feel that professionals do not approve of their beliefs and decisions, there is a risk that parents may choose alternative treatments that could be ineffective or even harmful for the child’s health and developmental outcomes. In ASD research, Kleinman’s conceptual framework has been widely applied in several studies in various cultural contexts, e.g. Australia [24], Canada [25, 26], India [27, 28], Kenya [29], and Taiwan [10]. To the best of our knowledge, the present study is the first in Sweden that uses Kleinman’s framework to investigate explanatory models of autism held by families of children with ASD from culturally and ethnically diverse backgrounds. In addition, it is the first study to date that specifically uses the Explanatory Model supplementary module of the core CFI to analyze the collected data.

### Sweden as a cultural context

Sweden presents a cultural context characterized by free universal child health care with routine screening for developmental delays and assessment protocols for diagnosis of ASD [30, 31]. Children suspected for ASD during routine screening at child health care centers (CHCCs) are referred to Child and Adolescent Psychiatric Clinics (CAPCs) for diagnostic assessment. Children with a clinical diagnosis of ASD are then referred to regional disability services known as child disability services, or child habilitation centers (CHCs), and enter state-funded intervention programs consisting of psychoeducational interventions for parents, and individualized or group-based interventions for children, such as speech and language therapy, occupational therapy, and treatments based on the principles of applied behavior analysis (ABA) [32, 33]. An important law that specifically regulates services and support provided to individuals diagnosed with ASD is the Act Concerning Support and Service for Persons with Certain Functional Impairments (1993:387) [34], known as *LSS* in Swedish. Under this law, children diagnosed with ASD and their families are entitled to eight services: (1) advice and other personal support; (2) personal assistance; (3) companion service; (4) a contact person; (5) relief service in the home; (6) short-term stay away from home; (7) short period of supervision for schoolchildren over the age of 12; and (8) living arrangements in a family home or in a residence with special services. Other laws regulating the provision of services to children with ASD are the Social Insurance Code (2010:110) [35], the Social Services Act (2001:453) [36], the Health and Medical Services Act (2017:30) [37], the Education Act (2010:800) [38], the Special Transport Services Act (1997:736) [39], and the Act on Housing Adaptation Grants (1992:1574) [40]. These services are provided to children with ASD for free regardless of their ethnic or cultural background, immigration status, or socio-economic status [41].

In the last few decades, Sweden has become a multicultural society, with migration being one of the main factors contributing to cultural diversity of the previously homogenous nation [42]. Data from Statistics Sweden [43] show that by the end of 2018, there were nearly two million (19.1%) foreign-born people residing in the country with a population of approximately 10 million inhabitants. Recent estimates of the prevalence rate of ASD in children were almost 1% [44]. Studies investigating risk factors for ASD have also revealed that a high percentage of children with the condition had both parents born outside Sweden [45]. Yet, there is a paucity of research concerning culturally and ethnically diverse families with children with ASD in relation to parental perceptions of the disability, patterns of interaction with service providers, and choice of intervention programs. Only one qualitative study conducted by Andersson [46] in Sweden has investigated multicultural aspects of ASD, involving the experiences of preschool and elementary school teachers who worked with multilingual children with ASD in specialized classrooms. The teachers reported that some parents were not willing to accept the diagnosis of autism as they perceived it as “the Swedish sickness”, something the child had “caught” in Sweden after having been regarded as healthy in the family’s home country [46, p.219]. The teachers also noted that it was difficult for them to know exactly how the parents perceived their child’s diagnosis, and wondered whether this was unique to immigrant parents who were not proficient in Swedish, or whether it might also be true for parents who were native Swedes. Although these findings offer a glimpse into the parental perspective, it is nonetheless paramount to obtain first-hand information from parents of this group of children. Knowledge about parents’ conceptualizations and explanations of autism and the onset of its symptoms can help professionals to better understand parents’ help-seeking behaviors and the decisions they make concerning intervention strategies for their child [47]. Knowledge about the reasons for parents’ treatment preferences can help professionals recognize how they can best support parents in their choices, and also guide them toward choosing evidence-based practices. It is hoped that in a long-term perspective, the insights gained from the present study could contribute to increased awareness among professionals about the impact of cultural factors on diagnostic assessment and treatment planning; and facilitate the development and provision of culturally sensitive, individualized interventions and services to meet the needs of each individual child with ASD and his/her family members in multicultural societies such as Sweden [48, 49].

### Aim and research questions

The aim of the present study was to explore parents’ explanatory models of their young children’s ASD within the multicultural context of Sweden. More specifically, the study sought answers to the following research questions:

1. How do parents from culturally, ethnically and linguistically diverse backgrounds recognize the onset of symptoms in their children with ASD?
2. What are the parents’ beliefs about the causes and mechanisms underlying their child’s autism?
3. How do parents seek help for their child, and what treatment decisions do they make after their children have obtained a formal diagnosis of ASD?

## Materials and methods

This qualitative study was part of a larger cross-cultural research project aiming at investigating parental experiences of raising a child with ASD and of interaction with support systems in the cultural context of Sweden. The study was exploratory and used a cross-sectional research design with a retrospective interviewing technique to collect data. The study was approved in 2015 by the Regional Ethics Board in Stockholm (2015/843-31/5). The ethics committee is now known as the Swedish Ethical Review Authority.

### Recruitment and participants

To participate in the study, the following inclusion criteria were applied: 1) families with various cultural, ethnic and linguistic backgrounds, including Swedish, who had young children (2–6 years old) with a clinical diagnosis of ASD based on either the ICD-10, the DSM-IV or the DSM-5 diagnostic criteria; and 2) families with an immigrant background had to have been living in Sweden for at least one year prior to participation in the study to exclude the possibility of post-traumatic stress disorder in young children exposed to migration-related trauma. The study used a combination of two sampling strategies to recruit eligible participants: purposive sampling [50] and network-based (“snowball”) sampling [51]. Thus, participants were recruited through: (1) regional CHCs; (2) professional networks (preschool teachers and special educators working with children with ASD); (3) parent support organizations; and (4) social media (Facebook). The consent forms and information letters describing the study’s aim, rationale, procedure, ethical aspects, and the researchers’ contact details in Swedish and other languages as suggested by CHC professionals (e.g., Arabic, Polish) were sent to contact persons to be distributed to parents of children with ASD. At CHCs, envelopes with a stamped return address were also provided. If the parents agreed to participate in the study, they were asked to send their written informed consent directly to the coordinating researcher (the first author of the study, with an educational background in special education). Before the study began, the researchers established relationships with parents via email or phone communication, when parents had the opportunity to ask questions about the study. In addition, representatives from parent support organizations acted as “gatekeepers” and helped establish rapport with parents. A final sample of 17 parents, representing 14 families from two counties in Middle Sweden and one family from the western part of the country, participated in the study. Written and verbal consent was obtained from all participating parents. Participants’ cultural and socio-demographic characteristics are presented in Table 1.

**Table 1:** Participants’ cultural and socio-demographic characteristicsª.

FamilyCode	Parent *(mother/father)*	Age *(in years)*	Parent’s Region of Origin	Time spent living in Sweden *(in years)*	Marital Status	Monthly Household Income in SEK (after taxes deducted)ᵇ
**F1**	Mother	31–35	Western Europe	Native Swede/born in Sweden	Divorced	Less than 20 000
**F2**	Mother	31–35	Western Europe	Native Swede/born in Sweden	Married	Less than 45 000
**F3**	Mother	41–45	Western Europe	Native Swede/born in Sweden	Married	Less than 35 000
**F4**	Mother	36–40	Western Europe	6–10	Married	More than 45 000
**F5**	Mother	36–40	Central and Eastern Europe /Caucuses	1–5	Married	Less than 35 000
**F6**	Mother	41–45	Central and Eastern Europe /Caucuses	21–25	Divorced	Less than 20 000
**F7**	Mother	31–35	Central and Eastern Europe /Caucuses	6–10	In partnership	Less than 35 000
**F8**	Mother	26–30	South America	11–15	In partnership	Less than 45 000
**F9**	Mother	31–35	South Asia	1–5	Married	Less than 35 000
**F10**	Mother	31–35	East Asia and the Pacific	1–5	Married	Less than 35 000
**F11**	Mother	36–40	Middle East and Northern Africa	6–10	Married	Less than 45 000
	Father	41–45	Middle East and Northern Africa	11–15	Married	
**F12**	Mother	41–45	Middle East and Northern Africa	1–5	Married	Less than 14 000
	Father	51–55	Middle East and Northern Africa	26–30	Married	
**F13**	Mother	26–30	East Africa	6–10	Divorced	Less than 14 000
**F14**	Mother	31–35	East Africa	6–10	Married	Less than 35 000
**F15**	Mother	26–30	East Africa	6–10	Divorced; in partnership	Less than 14 000

ªat the time of data collection.

ᵇIn Sweden in 2016, the yearly median income among single persons was SEK 172 000, and the corresponding median income among cohabiting parents with two children was SEK 257 000 [52]. Within the European Union, the at-risk-of-poverty rate is defined as “the share of people with an equivalised disposable income (after social transfer) below the at-risk-of-poverty threshold, which is set at 60% of the national median equivalised disposable income after social transfers [53].

Most parents were university educated (n = 10) with three of them holding Masters degrees; five parents had completed upper high school, and two had junior high school as their highest educational level. At the time of data collection, five parents were employed part-time, three worked full-time, one had a paid internship, three were unemployed, one was on parental leave, two were students, one was waiting for an internship, and one identified herself as a housewife. Parents’ religious affiliations included Muslim (n = 9), Christian (n = 3) and Jewish (n = 1), while four parents described themselves as “non-religious”. The majority of families (n = 12) had a boy diagnosed with ASD, and three families had a girl with ASD. One family had two children diagnosed with ASD. As reported by their parents, of the 16 children, eight were additionally diagnosed with intellectual disability (ID), and one child was diagnosed with social disorder. Of the 16 children, five were non-verbal, and four were minimally verbal (could speak some words or phrases). The average age of the children was 5.7 years.

### Instrumentation

Measures used in this study (as part of the larger project) include the Family Demographic Profile (FDP) [54] and in-depth interviews.

#### Family demographic profile

The Family Demographic Profile (FDP) is a questionnaire with structured questions used to elicit socio-demographic information about the child and the family members. The FDP is a modified version of the Parent Interview format used to investigate the parental experiences of adults with ASD in the context of India [54]. The original version consists of nine sections with structured closed and open-ended questions that capture various aspects of life for an adult with ASD and his/her family. For the purpose of the present study, only the first section of the instrument, titled “Background Information”, was used. The questions in this section were translated into Swedish and, where possible, adapted to the specifics of the Swedish context, e.g. for questions regarding family income, the name of the national currency was changed (i.e. Swedish crown).

#### In-depth interview

Semi-structured, in-depth interviews were conducted within the broader framework of the Ecocultural Family Interview (EFI) [55] which was used to address the aims of the larger research project: to explore the daily routines of families with children with ASD. The EFI is an assessment instrument defined as a free story-telling, or a conversation with parents of children with disabilities about the organization and sustainability of families’ daily routines and activities. [55]. The EFI is based on ethnographic interview traditions and usually begins by asking parents to “walk” a researcher or a clinician through a family’s typical day. To answer the present study’s research questions, some questions derived from the adapted version of Kleinman’s original set of questions [23] were integrated into the interview protocol in a slightly modified form. For instance, to elicit parents’ responses on the causes of ASD and treatment expectations, we asked the following two questions: *What do you think caused your child’s autism*? and *What kind of treatment do you think your child should receive*? (S3 Table). In order to capture one of the aspects of parents’ explanatory models–time and mode of symptom onset–we asked parents to recall when and how they recognized the first signs of their child’s condition before diagnosis, e.g. *Do you remember when you first noticed that something was unusual about your child’s behavior*? *When did you start suspecting that something was not right*? *Could you describe your child’s behavior*? To elicit the views of other family members on the child’s ASD, the following questions were used: *What do your relatives/What does your husband say about your son’s/daughter’s difficulties/problems*? Or: *What did you know about autism before it affected your child*?

### Data collection

Interviews took place between September 2015 and January 2017 at a place of the parent’s choosing: families’ homes (n = 8), public libraries (n = 3), cultural-religious centers (n = 2), the parent’s workplace (n = 1), or the local university college (n = 1). Interviews lasted between 1.5–4 hours. Two interviews were held with both parents present, and the rest with mothers only. All interviews were conducted by the first author in Swedish (n = 11), English (n = 2), and Russian (n = 2). During two interviews, an interpreter was present; in one interview, the father acted as an interpreter to enable his wife to participate in the study. Interviews were audio-recorded using a digital voice recorder.

### Data analysis

Reported data on the time of symptom onset was analyzed descriptively using the SPSS software program, version 24. The interviews were transcribed verbatim. To ensure integrity of the study participants and protect their identities, each family was assigned an alphanumeric code (e.g. F1, F2 etc.). Furthermore, the interview transcripts and audio files were stored in an external hardware device secured by an encryption software program [56]. Data were analyzed deductively using a directed approach to qualitative content analysis [57]. Five domains of the Explanatory Model supplementary module of the core CFI [1] were used as predetermined coding categories. Based on previous research on explanatory models of illness [17, 18] and on the definitions provided for each the five domains included in the Explanatory Model supplementary module [1, 19], the following operational definitions were used for predetermined coding categories to analyze data:

1. *Parents’ Understanding of Problem (Autism)*–how parents understand and describe their child’s condition before and after diagnosis.
2. *Autism Prototypes*–parents’ ideas about their child’s ASD based on the knowledge of others with the disorder, media coverage of ASD, or the parents’ own past experiences with a similar situation; the source(s) of information that shaped their understanding of the disorder.
3. *Causal Explanations*–perceived causes of the child’s ASD to determine how parents understand its source, reasons, and consequences.
4. *Course of Autism*–parents’ understanding of how the condition develops and what to expect in the future.
5. *Help Seeking and Treatment Expectations*–parents’ ideas about the most appropriate treatment, intervention and services for their child.

Using the operational definitions for each coding category, all interview transcripts were read through several times. While reading, different colors were used to highlight coding categories as described by Zakirova Engstrand and Granlund [58]. Analysis was also guided by the interview questions listed in the CFI–Informant version, Explanatory Models supplementary module (14 orienting interview questions). Some categories required further identification of subcategories. Each inductively emerging subcategory was given a relevant coding label (Table 2). At the last stage of the data analysis, all identified subcategories under each category were examined for shared patterns and similarities at various levels (e.g. events, processes, settings or time) using the clustering technique [59, 60]. Those subcategories that had similar meaningful characteristics were merged into clusters and generated themes (S1 Fig provides an example of the use of the clustering technique for the category “Causal explanations” across two time dimensions: before and after diagnosis of ASD). Thus, the themes were identified based on frequencies of subcategories and the results of the clustering technique (see Table 3; a more detailed description of the strategies used for data analysis is available upon request from the first author).

**Table 2:** Parents’ explanatory models of ASD: A meta-matrix of categories and subcategories identified during data analysis.

Categories and subcategories		Families															n*
		F1A	F2A	F3A	F4A	F5B	F6B	F7B	F8C	F9D	F10 D	F11 E	F12 E	F13 F	F14 F	F15 F	
**Parents’ Understanding of Autism**																	
	Symptom onset																
	• Early onset	x		x	x	x	x	x		x	x	x	x	x	x		**12**
	• Regressive onset		x						x							x	**3**
	Suspicions raised by preschool teachers			x	x										x		**3**
	Suspicions raised by child’s grandparents									x	x	x					**3**
	Father’s negative responses to mother’s suspicions													x	x		**2**
	Autism as an unfamiliar concept					x			xª		x	x	xᵇ	x		x	**8**
	Father’s reactions to child’s diagnosis	x		x		x	x	x						x	x		**7**
	Extended family reactions to diagnosis		x				x	x	x	x					x		**6**
	Explaining autism to others		x	x				x		x		x	x	x	x		**8**
	“Many have autism”				x		x		x				x	x			**5**
	Sharing only with those who understand										x			x	x		**3**
	“There is no shame about autism”								x				x				**2**
**Autism Prototypes**																	
	Family prototypes			x	x			x	x								**4**
	Comparison with typically developing siblings or other typically developing children	x	x	x	x			x		x	x	x	x	x	x		**11**
	Media prototypes (Internet, TV, satellite channels, books)	x			x	x	x		x	x	x	x	x	x	x	x	**12**
	Social prototypes (e.g. friends, work, neighbors)		x					x	x	x			x				**5**
	Self-prototypes			x	x												**2**
**Causal Explanations**																	
	Genetic/Hereditary		x	x	x	x	x	x	x	x			x				**9**
	Birth complications			x			x	x				x					**4**
	Congenital damage (at prenatal stage)							x	x								**2**
	Vaccinations						x		x						x		**3**
	Medication overdose					x	x										**2**
	Reaction to stem cell transplantation (causing ID)		x														**1**
	Reaction to gluten						x										**1**
	Reaction to GMO** food						x										**1**
	Vitamin D deficiency														x		**1**
	Supernatural/religious						x			x	x			x	x		**5**
	Reaction to parents’ separation or divorce	x					x									x	**3**
	Reaction to inadequate educational support (causing ID)											x					**1**
	“I don’t know”/”I cannot say”					x			x		x	x	x	x	x		**7**
	Exposure to several languages at home (and to Swedish at preschool)										x						**1**
	Reaction to physical abuse by peer at preschool							x									**1**
	Reaction to measles infection during trip to home country									x							**1**
	Head trauma (“fell down as a baby”)											x					**1**
	Cultural differences in childcare									x	x						**2**
**Course of Autism**																	
	Life-long disorder	x	x	x	x					x			x	x	x		**8**
	“Will be better”	x				x					x					x	**4**
	“Will recover”						x										**1**
	“We will see in the future”/”I don’t know”									x			x				**2**
	“Will develop and become like any other child”								x								**1**
**Help Seeking and Treatment Expectations**																	
*A Before diagnosis obtained*																	
	Formal help seeking in home country	xᶜ	xᶜ	xᶜ		x				x		x					**6**
	Formal help seeking in Sweden	xᶜ	xᶜ	xᶜ	x	x	x	x	x	x	x	x	x	x	x	x	**15**
	• Medical doctor/Family doctor		x	x		x						x					**4**
	• Pediatrician at child health care center								x					x	x	x	**4**
	• Nurse at child health care center							x	x	x			x	x	x	x	**7**
	• Psychologist at child health care center	x	x		x		x	x		x	x						**7**
	• Speech-language therapist	x					x	x						x			**4**
	• Specialists at child psychiatric clinics	x	x	x	x	x	x	x	x	x	x	x	x	x	x	x	**15**
	Special educational teacher at preschool			x	x												**2**
	Attending courses on parental skills				x												**1**
B. *After diagnosis obtained*																	
	Interventions/therapies obtained in home country	xᶜ	xᶜ	xᶜ				x									**4**
	Publicly-funded interventions and disability support services provided in Sweden	x	x	x	x	x	x	x	x	x	x	x	x	x	x	x	**15**
	Interventions/therapies obtained outside Sweden or home country						x			x							**2**
	Seeking private support and services in Sweden				x		x	x		x		x					**5**
	Lack of knowledge of ASD among teachers		x		x	x	x	x	x			x		x		x	**9**
	Lack of knowledge of ASD among primary health care professionals (nurses, family doctors)			x								x			x		
	Importance of early intervention	x		x	x		x				x	x	x	x			**8**
	Importance of personal assistant			x	x			x				x		x		x	**6**
	Importance of speech therapy	x						x	x		x	x	x				**6**
	Parents as mediators of children’s learning	x^e^	xᵈ^e^	x	x	x	x^e^	x^e^	xᵈ^e^	x^e^	x		x	x^e^	x^e^	x^e^	**14**
	Pharmacological interventions (melatonin)				x							x					**2**
	Complementary and alternative treatments			x	x		x		x	x						x	**6**
	• High dosage of vitamins C and D						x										**1**
	• Ayurvedic medicine						x										**1**
	• Dairy-free diet				x		x										**2**
	• Gluten-free diet								x							x	**2**
	• Sugar-free diet						x										**1**
	• Mineral water						x										**1**
	• Massage									x							**1**
	• Oil-based body lotions and creams						x										**1**
	• Hyperbaric oxygen therapy						x										**1**
	• Homeopathy						x										**1**
	• Elemental diet/food in liquid form			x	x					x							**3**
	Child listening to recitation of Quran															x	**1**
	Informal help seeking (friends, family members, social media)		x	x			x	x		x					x		**6**
	Asking God for help	x					x			x	x		x	x	x	x	**8**

*Note*:

*n–frequency of sub-categories across families.

**GMO–genetically modified

xª –In this family, the mother had some previous knowledge about ASD through her work, but reported a lack of knowledge on ASD in her home country.

xᵇ –In this family, the father had awareness of ASD, while the mother did not.

xᶜ - For this family, Sweden is their home country.

xᵈ - In this family, the father is also a “co-therapist”, i.e. both parents provide home-based intervention.

x^e^ - In this family, parents also use their own educational materials when providing home-based intervention.

A–Western Europe; B–Central and Eastern Europe /Caucuses; C–South America; D–South Asia; East Asia and the Pacific; E–Middle East and Northern Africa

F–East Africa

**Table 3:** Parents’ explanatory models of autism before and after diagnostic assessment: Categories, sub-categories and themes.

Categories	Before diagnosis	After diagnosis
**1. Parents’ understanding of child’s autism**	• First suspicions and time of symptom onset	• Autism as unknown concept • Explaining to others Autism as worldwide phenomenon
	• Family members’ responses to ASD symptoms and diagnosis	
**2. Autism prototypes**	• Typically developing children prototypes • Media prototypes	• Media prototypes • Family prototypes • Self-prototypes • Social prototypes
**3. Causal explanations**	• Child’s condition • Child’s reaction to external environmental influences	• Definite causes • Possible causes • Unknown causes
**4. Course of autism**	-----	• Lifelong disorder • “Will recover” • “Will be better”
**5. Help seeking and treatment expectations**	• Seeking help and information from health care professionals • Seeking help from non-medical professionals	• Importance of early intervention • Parents as mediators of children’s learning • Unmet expectations • Use of complementary and alternative medicine • Asking God for help

Several strategies were used to ensure the quality of the analyses [61]. Dependability of the study results was strengthened by check-coding [60]. To ensure consistency and stability over time for data coding, the first author initially coded the transcribed text of one interview (23 single-typed pages), and then later check-coded the transcribed texts of the remaining interviews. In addition, the first author and the last author (PhD-level researcher) independently coded three interview transcripts (≈ 20% of the material). Before initiating the coding process, both researchers met and discussed the operational definitions of each category as well as agreed on emerging sub-categories based on one interview transcript. After coding, differences in coding were discussed by both coders/authors until consensus about subcategories was reached. Following this discussion, the first author revised and, where necessary, re-coded the interview transcripts. The inter-rater reliability for two coders was also calculated using percentage of agreement. Percent agreement was 0.86. Thereafter, the study’s findings were discussed with the rest of the research team. To address credibility of the findings, the interview transcripts and summary reports were sent to participants for verification. In addition, external auditors (professor-level researchers) assessed the accuracy of inferences drawn from the study results on several occasions. To enhance transparency of the findings, data on frequencies of subcategories across the families are presented in a matrix format (Table 2).

## Results

The presentation of the results is organized around five aspects of explanatory models (see above), and for some aspects across two time dimensions: before and after a diagnosis of ASD was obtained (Table 3). To ensure confidentiality of data and anonymity of the study participants, quotations are not matched to the participants’ demographic information. (Due to richness of the results of the study, we have chosen to report only the most interesting findings here; more comprehensive results of the study are available upon request from the first author).

### Parents’ understanding of autism

#### First suspicions and time of symptom onset

Parents reported the time of symptom onset for their children as ranging from six months to 36 months old (*M* = 18.81, *SD* = 9.68), with the majority of parents reporting symptom onset before the child reached the age of three (n = 12). Some parents noticed the first symptoms during the child’s first year of life. These parents described their child’s behavior as unusual: children did not respond to their names, and did not say “mom” and “dad”; some of them did not make eye contact. Other frequently mentioned symptoms included sleeping problems and tantrums. Three parents described their children as developing very well at first, but as losing acquired skills later on. For instance, one mother recalled:

He was an active child and functioned very well. He was very nice. And when he was approximately 2 years and 7 months old, he became forgetful, he started losing his speech. <…> I saw that he started being selfish … being by himself and didn’t want to play with other children he used to play with before.

Many parents noted that they started suspecting something was wrong when either friends or extended family members, such as the child’s grandparents, began asking questions about the child’s developmental milestones, e.g. “Has he started talking? Why is he not talking?” One mother from an immigrant background described how her own mother expressed concerns about her grandchild’s development: “I didn’t notice anything. I noticed when he was 1.5 years old after my mother said to me, ‘You need to check him’.” A Swedish mother said: “When my parents heard my description [of the child’s disruptive behavior] and witnessed it once, they said that it shouldn’t be like that. Then I decided to seek help as none of the methods I tried, worked.” Other parents reported that their initial suspicions were confirmed by those raised by teachers based on their observations of children’s behavior at preschool–for instance, as one mother recalled:

One day it was the father who left [*the child’s*] at the preschool … and there was a preschool teacher, an old [*lady*], who said to the father: ‘I believe your child has autism. I have worked with children with autism for 20 years’. He came home, opened the door, and he was crying…

#### Family members’ responses to ASD symptoms and diagnosis

Mothers described sharing their initial suspicions with others family members, mostly spouses. Fathers’ responses to first signs of autism or to an ASD diagnosis ranged from being reluctant to view their child as with ASD to very negative reactions to mothers’ concerns. For instance, one mother described her partner’s initial reluctance to accept their daughter’s ASD diagnosis in the following way:

We decided to accept the diagnosis … Besides, [*the name of the partner*] was not really… I mean, he didn’t immediately agree with the diagnosis … He didn’t accept it. He accepted it later [*after*] I talked to him, [*after*] the psychologist talked to him. He was given two weeks to think about the diagnosis–whether to accept it or not. He accepted it, and then the diagnosis was given.

In another family, a mother reported that her child’s father had said that there was nothing wrong with the child, he just had insomnia. This father mostly blamed the mother for her having a “psychiatric imbalance” and strongly advised her not to tell anyone about her suspicions. In another family, the father’s reaction to the mother’s suspicions was anger. This man had lived in Sweden for 20 years and had heard about autism. The mother recalled: “He got a bit angry with me. [He said]: “Why are you saying this? You’re a mother and you are saying this kind of thing about your own daughter! Why? You’re saying something that is not good.” In another family from an immigrant background, the mother described her own and her ex-husband’s different views about the child’s condition, referring to their different cultural backgrounds:

We’re very different … I’m integrated into [*Swedish society*] <…> but he can’t do that. He still has his beliefs [*from his region of origin*]. He believes that if I am doing this [*get help for the child’s condition*], then I am manipulative to make him come back to the family. <…> He didn’t believe me [*about the diagnosis*]. He blamed me … that I made it up so that I could skip working and take care of the child instead … He has a girlfriend who works as an assistant nurse at the [*name of the hospital*] who once looked at the child’s photograph and determined that he [*the child*] is totally normal. So he blamed me …

However, regardless of cultural and ethnic background, the majority of mothers did not mention that their husbands blamed them for their child’s autism. In fact, in one family from an immigrant background, it was the father who first noticed the onset of ASD symptoms in his child and drew the mother’s attention to this.

Study participants also described the reactions of extended family members to ASD symptoms and diagnosis. For instance, some mothers from an immigrant background stated that their own parents, and in the case of one family a grandparent, initially viewed the child’s unusual behavior as a result of “bad upbringing”; for instance, one mother said: “My father and his wife cannot understand that–they are old people, in their 70s. They believe that if you spank [the child] on his buttocks, then he will be in order.” Other close relatives reacted with sadness; one mother recalled her elder sister’s response to her nephew’s diagnosis:

She felt bad because it was something we hadn’t heard about … or we *did* hear about it but we never believed that something like this could affect a close family member … She felt bad … Her own children talk and they are independent … she couldn’t understand why it was *my* son who [*got autism*] … She didn’t know how she could help me [*crying*].

#### Autism as unfamiliar concept

In response to interview probes to elicit the family’s prior understanding of ASD before their child showed first signs or received a formal diagnosis, some parents with a background from non-Western societies reported that they had never heard of autism before they came to Sweden. As one parent stated, “As soon as one comes to Sweden, one hears about this diagnosis!” Several mothers admitted that they had never come across the word “autism”. One father who had lived in Sweden for 28 years also reported his unfamiliarity with autism, summarizing succinctly: “Many [parents whose children] received [the diagnosis] didn’t know about this autism.” Some parents who came from East Africa described strategies they had used to understand the concept of autism, for instance:

I didn’t recognize autism, this word “autism”… in Latin … I didn’t recognize it <…> I watched a program in Arabic, and there was a doctor who sat there. They talked about autism. But this word “autism”… in Arabic it doesn’t sound like “autism”, they call it “Altawahud” <…> “Altawahud” and “autism” are the same. I reflected [*on this*] immediately. I started searching for “autism”. I went to YouTube and wrote [*the word*] in Arabic, and I checked it: those who have this problem, [*they*] have autism.

#### Explaining autism to others

The more parents learned about the disorder from the day they started suspecting, and especially after their child obtained a formal ASD diagnosis, the easier it became for parents to explain their child’s condition to others in the local community. For instance, a Swedish mother said she felt it was important for her to explain autism to other children and their parents at her child’s preschool: “The best way to deal with prejudice is to know that everyone is different.” Mothers from an immigrant background reported that their close relatives who lived either in Sweden or outside Sweden did not know about ASD and therefore asked many questions about the child’s condition. For instance, as two different mothers recalled:

… They [*mother’s parents and parents-in-law*] did not have any information about autism. They did not know what autism is. So we had to tell them about it. It was very strange for them. They said: “We have not heard about this before,” and then we explained to them what it is.

[*… the relatives*] ask many questions like “Is he crazy? Does he understand nothing? What is it with him? Does he meet with other children?” <…> My siblings and other who are saying this … This [*autism*] is something they don’t know about.

### Autism prototypes

#### Typically developing children prototypes

Parents said that their suspicions about their children’s unusual behavior led them to compare their children with other typically developing children of the same age. Some parents reported asking other parents about their children’s developmental milestones (e.g. “I asked other parents about how a child of this age [should] behave”). Parents whose first child had been diagnosed with ASD made retrospective comparisons with their unaffected children (the child’s younger siblings), e.g. as one mother described:

They [*the children*] are like two different individuals. When [*the name of the younger son*] was born, I could feel the difference … it was like day and night. He [*the younger son*] has always been so close to me, so sweet and cuddly, so much tied to me–all of this that I felt that I’ve never really had with [*the name of the son with ASD*].

#### Media prototypes

Various types of media were the most commonly reported sources of information that shaped parents’ understanding of autism and caused them to make analogous comparisons to their own child’s atypical development. Most families reported using books or the Internet to search for information. As one father revealed:

During Christmas celebration we noticed that something was not right with the child. He was 2 years and 4 months old, and he did not at all react to Santa Claus, or the presents; he even did not look at us–he was not present. <…> When we came back [*home from celebration*], I started reading a lot about “unresponsive kids”, about everything [*the child’s name*] was doing, and I saw all the symptoms pointing to autism.

Other families from an immigrant background reported watching satellite television programs broadcasted in the parents’ first or second language, and programs on Swedish television. For instance:

I watched a program in Arabic <…>. Several children who were sitting there [*with their*] parents, and the doctor … they were talking about autism. I looked, I heard [*that*] all of them had a diagnosis. They said the same thing about my child … They are the same! She [*the child*] has the same as those children. <…> These children had the same eyes, they didn’t have eye contact … the same face and the same problems … they had also problem with speech <…> Then I watched a TV program in Swedish. There was a film, where they were talking about a girl who had autism, about a family that had difficulties; she could not talk.

For some families from an immigrant background, media continued to be an influential factor in shaping their understanding of autism after the child was formally diagnosed with ASD. For instance, as one parent said: “Later I tried myself to search YouTube, the Internet, and asked other parents who had children with autism, and I got lots of information and knowledge about the diagnosis.” Another parent reported:

I started reading on Internet; I watched all the movies on YouTube, and one of them was exceptional: “Rain Man” with Dustin Hoffman and Tom Cruise. I also watched a film with … Temple Grandin … and I realized that not everything is so scary; that it [*autism*] is not a disease; it is not a verdict. It is a way of life.

Parents’ narratives about their child’s condition after obtaining the formal diagnosis also revealed that their awareness of ASD was based on prototypical experiences of self and of others–either family members or people in their social network.

#### Self- and family prototypes

Some families, especially those of a Western or Eastern European background, related their child’s problem to other members of the family, e.g. a cousin or uncle, who had experienced a similar condition, either diagnosed or undiagnosed. Typically, these families connected family prototypes to genetic factors of autism etiology. For instance, in one of these families the child’s condition was related to the father’s self-prototypical experiences:

I believe that … well, my … [*name of the husband*]’s sister’s child has it. And as soon as one recognizes the problem, then one can see that there are several people in the family… and my husband probably also has some small characteristics of [*the condition*] <…>. But he doesn’t have any diagnosis … but he himself also believes that it is a little bit … he recognizes some traits so to speak … So I am quite convinced that this is purely genetic.

#### Social prototypes

Several families reported that they had met children in their social environment who experienced similar problems to their own children; for instance, three families had earlier or current work experience with children with ASD in Sweden. Two other families talked about neighbors who had a child on the autism spectrum. One father said of his wife’s experience:

His [*the child’s*] mother knows a lot about [*autism*] because she reads a lot on Internet. In [*name of the home country*] she had a neighbor, a boy … He’s very angry. He wants [*at mealtime*] to have a specific glass; he doesn’t want to change the glass. The food that he eats, the plate, the glass or … he wants the same. He doesn’t want a change.

### Causal explanations

#### Initial causal interpretations: child’s condition and reaction to external environmental influences

Parents reported that initially, they attributed their child’s behavior to some other medical diagnosis or condition (see Table 3 and S1 Fig). For instance, some parents suspected that it was hearing loss that caused their child not to react to parents’ voices or respond to his/her name; other parents thought that their child’s screaming was due to colic; some other parents explained their children’s seizures as epileptic (an initial medical diagnosis for two children in the present study). For instance, one mother said:

When we came here [*to Sweden*] … we went to the hospital with the ambulance as he [*the child*] had seizures during that night and he cried so much-much-much-much-much … and they [*doctors*] saw it … I couldn’t speak Swedish, I was an asylum seeker … I said [*that*] my son has seizures, but not in Swedish. I just said “epilepsy, epilepsy”.

For three mothers in the study, tense relations with spouses and going through a divorce were other suspected causes of the child’s problem behavior, e.g. “His [the child’s] father and I got married and then shortly after we got divorced… I was worried a lot about it, and I thought that maybe my worries passed on to him.” Parents also described noticing changes in their child’s development after a specific event or activity that had directly affected the child. For instance, one mother shared:

He went through [*the procedure of*] stem cell transplantation when he was one year and a half. And till that age we thought he was developing normally. … But after stem cell transplantation … he stayed in the hospital for a long time, and when he was there I noticed that he started isolating himself from the nurses. He stopped looking at the doors and didn’t look in the eyes.

Two mothers from South-East Asian countries believed that cultural differences between childcare in Sweden and in their home countries could be responsible for their child’s developmental delays:

You know, in our country we are brought up in very big families; there are many kids together and they [*children with developmental delays*] just grow up together with the other kids and, eventually, they start learning by themselves. And we never notice that they have problems. But here, it’s more like, you know, isolated. And we just ourselves in this home–we don’t have my parents, my husband’s parents. Usually, in our country we have our parents’ grandfather living together. So, there are more people communicating and have interaction. So, they [*children with developmental delays*] just grow up and eventually they become better in social communication. <…> But here we notice it so much because we are living in a small family; we don’t have anyone and he is our first son, he doesn’t have any siblings, so he has only us. We thought: he is delayed because of this kind of thing.

#### Definite, possible and unknown causes

Data analyses revealed that parental explanations for their child’s ASD after diagnostic assessment could be grouped into three main clusters: *definite causes*, *possible causes*, and *unknown causes* (S1 Fig). The most frequently cited explanations in the first group were heredity or other genetic factors. Vaccinations were also mentioned as a definite cause for their child’s ASD. As one mother said: “I am 100% sure that something happened [to the child]. And I’m saying it was due to vaccination. I read a lot… You know, these vaccinations can lead to brain inflammation.” Among other frequently believed causes for the child’s ASD were of a supernatural or religious nature. For instance, several mothers interpreted their child’s condition as a blessing or a gift from God:

I feel it’s a blessing. I just feel that Allah always tests very near people, so I feel that I am blessed that He has chosen me to be his [*the child’s*] mother. And it is an honor to me because I know that there was something that He chose … that I could do that nobody else could do, that He chose me to have this challenge because nobody’s life is perfect. Everybody has to face something different in life.

Among possible causes, parents mentioned several environmental factors that could potentially have contributed to their child’s condition, such as reactions to gluten or genetically modified food, high dosage of medications, and vitamin D deficiency; others included birth complications and physical trauma. Many parents said that they simply did not know the exact reasons for their child’s disorder, although they recognized that it could be related to genetic factors, i.e. something that runs in the family. For instance, as one father said: “I don’t know anything. I don’t know why. We didn’t have anyone in the family [with this condition].”

The analysis also revealed that families generally had more than one possible explanation for their child’s disorder. For instance, one mother explained her child’s disorder differently on two separate occasions:

We [*the family*] believe into several things about [*the cause*]. At the beginning they [*the relatives*] asked me: “Did you do anything to your husband’s mother? Anything? Maybe you said something [*to mother-in-law*]?” No! But they believe so, they believe so. They say:”You said something bad to other [people], that’s why your child became like this.”

And later:

I heard that … doctors on YouTube … in Arabic … this doctor said that it happened to several families whose children were vaccinated. I just heard that, I am not sure about anything. In Sweden they say “no” but in other countries they say “yes”. And the problem is not in vaccines themselves, the problem is in metals that don’t disappear <…> Vaccines have substances that protect them from [*spoiling*]; when the date expires, these substances that protect vaccines get destroyed and they cause problems for children. The doctors abroad say so. And they also say that if we don’t vaccinate children before age of three, they don’t get autism. <…> In my heart I believe in that. This was first. The second, it can happen that we have low [*levels*] of vitamin D as we have much clothes and we are protected from the sun, so this can happen, they say here in Sweden, the doctors, they believe so. They are not sure but they believe so. They believe also that due to this there are many who have autism among us, [*name of ethnic group*] people.

### Course of autism

Parents shared their views on the prognosis *after* their children obtained a clinical diagnosis for autism and entered intervention programs. The majority of parents believed that autism was a lifelong disorder. Parents from Western European countries explicitly stated that ASD was a dysfunction or a disorder that could not be cured, although the difficulties could be reduced: “It doesn’t feel like this disorder is going to be cured, but I believe … well, [name of the son] has learned to express feelings, [although] I am totally convinced he cannot really feel them.” The belief that ASD is a lifelong condition was intensified for one parent whose son was also diagnosed with intellectual disability:

When [*diagnostic assessment*] was finished, and he got his diagnoses, it felt like … well, we had been [*mentally*] prepared for autism in any case, but intellectual disability … it was the hardest… that he also got intellectual disability… because it does mean a more comprehensive disability for the rest of [*his*] life.

Many parents from a non-Western cultural background were cautious about giving any prognosis due to their child’s young age (e.g. “We will see in the future”). These parents expressed hope that their children’s condition would improve and that they would be able to learn necessary skills with time. For some families, mastering language skills was an important prerequisite for the child’s positive development and for prognosis:

We cannot judge on [*name of the son*] as he is little. You cannot say where he is now, on what level. I think when he starts talking, then one [*could*] judge on where he is. You know, he develops all the time … Maybe in the future he will be better and better and better … But most of all, language is very important for him.

Other families thought that their children would develop and “become like any other child”, i.e. believed in their children’s recovery from autism.

### Help seeking and treatment expectations

#### Help and information from health care professionals

All parents regardless of cultural background reported that actively seeking help and information from health care professionals in Sweden had been their major effort to understand their child’s unusual behavior. All parents except for one mother reported turning to local Child Health Care Centers (CHCCs) to discuss their suspicions with nurses, pediatricians or psychologists, who in turn referred children and their families to regional Child and Adolescent Psychiatric Clinics (CAPCs) for assessment and confirmation of an ASD diagnosis. For some families coming from regions other than Western Europe, seeking help from medical professionals in their home countries was a viable and important possibility to get a “second opinion”. This was especially true for those families for whom there were no restrictions on travel back to their home countries. One mother, for instance, described her thoughts and feelings in the following way:

<…> because in Sweden you cannot get the second opinion–it’s just one person telling you; then you cannot choose your doctor here. In [*name of the home country*] you can choose your doctor and I know he was the best doctor. He was a doctor since I was a child and … I really trust him a lot. It’s not that I don’t trust the system here, I definitely do, but … I kind of felt that I needed the second opinion.

#### Seeking help from non-medical professionals

Some families, primarily those representing Eastern and Western European countries, said that before they decided to share their concerns with CHCC staff, they had tried to use other strategies to address their child’s problems. For instance, two families reported turning to speech therapists to get help for their children’s speech delays; one family reported attending various courses to learn about effective parenting strategies regarding young children, although these were not found to be very helpful:

He [*the child*] has severe tantrums sometimes … and we thought it was normal, but now we believe it wasn’t. But we [*thought*] we should do something about it. <…> There is a course for parents to children aged 0–3, and there is [*a course called*] Komet … but it became just worse and worse.

#### Help seeking and treatment decisions after diagnosis was obtained Importance of early intervention

Parents emphasized the importance of early intervention for their children so that they could develop in the optimal way and acquire necessary skills. As one mother put it: “For children with autism, one must think early.” A father noted: “Every second, every day, every minute means a lot for autistic children! Time is not their friend!”

#### Parents as mediators of children’s learning

The analyses revealed that almost all interviewed parents emphasized the importance of teaching social, communication and play skills to their children at home. Parents (especially mothers) reported that it was their highest priority to teach and train those skills by using symbols, pictures, toys, drawings, books, play and role-plays. Many parents described teaching sessions with their children as being embedded in their everyday routines such as brushing teeth or getting dressed, e.g. “He [the child] seldom puts on his shoes by himself. He says, ‘Help me’–and this is what we also taught him to do–to know how to ask for help.” Some parents also reported that they used other resources (e.g. parents’ workplace or the Internet) to obtain educational materials, or created their own materials for teaching social and communicative skills at home. For instance:

I got some [*the pictures*] from my colleague … But I thought they were boring as they were not colored. How can children learn from them? So I made the pictures and hung them on the fridge–weekdays–in different colors. When it’s Wednesday, then it should show that he [*the son*] is going to take the bus, i.e. “the bus day”…

#### Unmet expectations

All interviewed parents reported obtaining various types of disability support services for their children. However, while for some families this was a real help, for other families getting a personal assistant for their child became the most challenging endeavor, especially if the child was placed in a general preschool setting. Many parents complained that general preschool teachers lacked knowledge about how to meet the needs of children with ASD, for instance:

When [*my son*] had an outburst, I noticed that they [*preschool staff*] didn’t know how to manage him <…> They must have one person who would know how to handle such situations … because a preschool teacher cannot handle this even though [*they hold*] a teacher certificate … I think there should be a right person for those kids with the diagnosis.

Some families felt that it was not the municipality but them who had to navigate all the complexities of service provision, which was stressful for parents:

… It [*relief service*] doesn’t work, it doesn’t work at all… because it is an 18-year old girl who is sent to us and who must build up relationships during three hours per week. This creates more stress than anything else, only stress. And then they don’t know what they’re going to do, and, therefore, an evening before we ourselves must do preparations and think over what she could do with him [*the child*], something that he likes doing.

Teachers’ lack of knowledge coupled with municipalities’ rejection of applications for personal assistants, or being delayed access to other services, caused some parents from an immigrant background to feel very frustrated with the public support system and to seek out private services. However, financial constraints hindered some immigrant families from obtaining private special educational services: “I would like a special teacher to give lessons to my son… if it’s possible to find one. There are some special teachers who charge a fortune: 12 000 SEK for 10 lessons. It’s very expensive…” On the other hand, immigrant families that were financially better off reported having difficulty locating private support services: “For a long time we looked for a person who would specialize in this kind of children, who would understand them and help us… but we couldn’t find one.”

Some families reported their dissatisfaction with support provided by child habilitation centers. Parents felt that interventions were not as timely or intensive as they had expected. Parents’ needs being unmet led them to look for alternative ways of obtaining intensive one-to-one interventions for their children. For instance, one family unsuccessfully tried to get in touch with a private clinic offering an early intensive behavioral intervention (EIBI) program to young children with ASD. Other possibilities that families considered were to return to their home countries to access interventions. However, for some of the interviewed parents who made the journey, those interventions were too costly, and they had to travel back to Sweden where the child was placed on long waiting lists for interventions. As one mother reflected:

… It takes time for them [*professionals in Sweden*] as they have too much to do … And I think like this: … well, here [*in Sweden*] it doesn’t cost anything, but in [*name of the home country*] it costs money. If someone has a child with [*autism*], it is us, parents, who must pay for interventions provided at their habilitation centers. If one needs to go to a speech and language therapist, it is us, parents, who must pay; if one is going to an occupational therapist, one should pay for everything. But there they [*children with autism*] get various kinds of support from various … [*professionals*]. And one wonders: why don’t they get support in the same way here [*in Sweden*]? <…> Here I could get only a speech therapist, but I know that one can get [*other kinds of support*]. I don’t know if they are available here, in Sweden.

#### Use of complementary and alternative treatments

Some families reported the use of complementary and alternative treatments for their children alongside interventions obtained at the CHCs (Table 2). These treatments ranged from nutritional supplements (some prescribed by physicians) and dairy- and gluten-free diets to massage, hyperbaric oxygen therapy, and homeopathy. For instance, one parent noted: “I give [the child] a big dosage of vitamin D; he also takes vitamin C in big quantities. Soon we will start taking zinc and selenium.”

#### Asking God for help

Several parents, irrespective of their religious affiliation, said that they prayed to God to help their child. One mother revealed: “My prayers come from the bottom of my heart, and this prayer is usually the Christian Orthodox prayer–a prayer of the Holy Mother of God, for her child.” In addition, for many parents talking to God was a relief; for some, God was the only shoulder to cry on. For instance, one mother said, when asked who she turned to in difficult moments of her life: “I think about God and try to ask God for help [to get] relief in my situation. I try to avoid telling other people about my secrets.”

## Discussion

To the best of our knowledge, this is first study to use the CFI framework [1] to investigate parental explanatory models of their children’s autism in the multicultural context of Sweden. Fifteen families from diverse cultural, ethnic and linguistic backgrounds were interviewed about their recognition of young children’s ASD symptoms, perceptions of the causes, help-seeking strategies and treatment choices, revealing both similarities and differences in regard to ethnicity and country of origin. The main findings of the present study are discussed below.

### Parental recognition of ASD symptom onset

The results indicate that regardless of cultural or educational background, parents of young children who would later receive a diagnosis of ASD were able to notice and report first signs of autism to primary health care professionals at the average age of 18.81 months, with some parents reporting first signs as early as six months of age. This finding is consistent with previous results reported by Becerra-Culqui et al. [62] showing that parental concerns arose at an average age of 18.1 months for children diagnosed with ASD before the age of three. Furthermore, the results of the present study indicate no large differences in types of parental concerns based on parents’ ethnicity/country of origin: families reported similar types of concerns, with the most frequently reported being speech and language delays, lack of social interaction/communication, and lack of eye contact. This result is in line with previous research on culturally diverse families with children with ASD which did not find differences in ASD-related developmental concerns based on families’ ethnic or racial background [63]. In addition, we did not observe differences in types of parental concerns based on parents’ level of education. Instead, our data indicate that there were other factors that may be accountable for parents’ recognition of early signs of autism, such as environmental factors and parents’ knowledge and experience (or lack thereof) with typical developmental trajectories. For instance, in some families, key figures in the child’s proximal environment such as preschool teachers raised concerns and drew the parents’ attention to potential symptoms of ASD. Interestingly, a recent Swedish study by Nilsson Jobs et al. [64] demonstrated that preschool teachers were able to rate ASD symptoms in high-risk children with and without ASD diagnosis more accurately than parents, as teachers usually have the opportunity to meet a wide range of children, which allows them to compare children’s behaviour in a more normative way. Similarly, in some families, grandparents’ queries about their grandchildren’s developmental milestones triggered parents’ concerns and led them to seek formal help, especially among those parents who lacked experience of children’s typical development trajectories. To compensate this lack of experience or knowledge, parents employed various strategies to understand their children’s unusual behavior, with the most commonly reported–coined here analytically as *typically developing children prototypes*–being to compare their children’s behavior with the children’s typically developing peers or siblings.

Previous research suggests that early recognition of autism greatly depends on parents’ knowledge and awareness of ASD [65]. The results of the present study show that for many immigrant families from geographical regions outside Northwestern Europe, “autism” was an unfamiliar word. This finding echoes the results of previous studies conducted in the UK [66, 67] with families of children with ASD from a Somali background, who highlighted the absence of a Somali word for autism. Our data show similar results for families from several non-Western cultures, who revealed that they had never heard the word “autism” before they came to Sweden. This finding can be explained by several factors. For instance, it can be argued that the term “autism” could be considered as a concept inherently rooted in a Western biomedical tradition. The evolution of the concept can be traced back to the Swiss psychiatrist Paul Eugen Bleuler, who was first to use the word “autism”, derived from the Greek word αύτός meaning “self”, to describe behavioural features among individuals with schizophrenia [68]. Later, the term appeared in two diagnostic systems: the DSM and the International Classification of Diseases (ICD) [69]–also essentially Western medical tools that largely reflect European thinking [70–72]. However, despite the fact that the World Health Organization (WHO) included “autism” in its classification of psychiatric and developmental disorders several decades ago, in some developing countries the notion of autism is still largely unknown to many medical or health care practitioners [73, 74]. Several authors argue that in many low- and middle-income non-Western societies, the field of child psychiatry is only in its infancy, which may leave many children with ASD or other developmental disabilities undiagnosed and without treatment [73–76]. Moreover, in some traditional societies individuals with mental illness or disability may be viewed as being possessed with evil spirits or affected by other supernatural forces such as curses or witchcraft [29, 77]. Such cultural beliefs may result from a lack of knowledge about ASD and inevitably lead to the stigmatization of children and their families in their communities [19]. For instance, research from non-Western societies has shown that on the societal level, parents may be blamed for their child’s autism [29].

The results of the present study also show that in Sweden, some parents from an immigrant background became familiar with the notion of “autism” and obtained a basic understanding of ASD through various types of media, primarily the Internet and television in parents’ first or second languages (*media prototypes*). Generally, the results indicate that parents’ prior knowledge of ASD seemed to be influenced by several factors: (1) parents’ previous experience with children with autism through work or their social network (*social prototypes*); (2) being born in Sweden or another Western European country; (3) residing in Sweden for a long period of time, for families who had emigrated from regions outside Western Europe (i.e. the longer they had lived in Sweden, the more they knew about autism); and (4) family history of autism (*family prototypes*). These findings could be explained by the biomedical view of autism prevalent in Sweden, as well as the higher level of public awareness about ASD in Western societies, including Sweden, than in non-Western countries [78, 79]. Indeed, research from several developing countries has shown either a lack of knowledge [80] or very low awareness of autism among the general public [81–83].

On the other hand, the results suggest that other factors might also affect parents’ prompt recognition of their child’s atypical behavior indicative of ASD. For instance, the results point to gender differences in appraisal of the child’s ASD symptoms, which is consistent with previous research with immigrant families in the multicultural contexts of Canada and the USA [25, 84]. As our findings show, in some families from an immigrant background, mothers detected possible developmental problems in their children and applied various strategies to understand atypical behavior, while some fathers expressed denial or anger and were less accepting of their children’s condition, despite being aware of autism or residing in Sweden for a long time. One explanation for fathers’ negative perceptions of ASD could be societal attitudes characterized by shame and stigma towards children with disabilities found in some non-Western cultures [85, 86]. However, this result and interpretation should be approached with caution for several reasons. First, our study showed that some fathers with a non-Western cultural background did not hold negative perceptions of their child’s ASD, and reported acceptance of their child’s disorder and no feelings of shame. Second, the results also showed that in two Swedish families, fathers did not entirely agree with mothers about their child’s diagnosis and/or interventions. These findings could be indicative of existing gender differences in parents’ attitudes to early screening for ASD, and perceptions of diagnosis and interventions irrespective of parents’ cultural background, echoing the results of previous studies in the field [87]. Another reason why these results should be treated with caution is that in this study, fathers’ experiences and reactions to early screening and diagnosis were reported predominantly by mothers. Further research is necessary to shed light on the apparent complexity of these findings, especially given the fact that research involving fathers of children with ASD is scarce [87, 88]. Future studies could investigate differences and commonalities in perceptions of ASD among fathers from various cultural backgrounds, and identify factors affecting their views in the context of Sweden and other multicultural societies.

### Parental beliefs about the causes of ASD

The results demonstrate that genetic factors were the most frequently believed cause of autism for many parents included in this study, which is congruent with the results of previous studies [10, 24]. To date, evidence from genetic studies on autism, including twin and family studies, shows that genetic factors can contribute to the development of ASD [89], and that ASD can overlap with other neurodevelopmental disorders sharing risk genes, resulting in the manifestation of signs and symptoms of autism in such syndromes as tuberous sclerosis, fragile X syndrome, and Rett syndrome [90]. The results demonstrate that almost half of the families (n = 7) could not say with certainty what exactly had caused autism in their children, which is consistent with the findings of a recent Canadian study conducted with immigrant families [25]. However, the results revealed that the majority of parents provided multiple explanations for their child’s autism, which supports findings from previous studies [10, 24, 28]. There are several possible interpretations of these findings. On the one hand, these results can be seen as reflective of the current state of research on identification of the causes of ASD, which suggests a complex interplay of both genetic and environmental factors potentially contributing to ASD in young children [89]. On the other hand, the results demonstrate that parents’ multiple causal explanations for their child’s ASD are closely linked to information gained from a variety of sources, illustrating the significance of social contextual factors influencing the development of explanatory models [17]. For instance, parents in the present study might hold various explanations simultaneously depending on a) the views and interpretations of extended family members, b) information obtained from health care professionals, and/or c) information obtained independently through various media. In addition, for some families from an immigrant background, beliefs about certain causes were dominant and preferable over others. Furthermore, the analyses showed that parents’ causal beliefs about ASD were influenced or confirmed by various media in parents’ first or second languages (i.e. other than Swedish); however, messages conveyed by those media might not always be in agreement with current biomedical knowledge in use in the Swedish health care system. This is an important observation, as parents might have obtained information that is not supported by current scientific evidence. For instance, previous research has indicated that media coverage of the theory that vaccines are a risk factor for ASD significantly influenced parents’ causal beliefs about autism, and subsequently their decisions about vaccinating their children [91]. The biomedical view on ASD etiology rejects this theory, which is supported by the latest evidence demonstrating no association between the measles, mumps and rubella (MMR) vaccine and autism [92]. In the present study, three parents from an immigrant background representing various geographical regions (Eastern Europe, East Africa and South America; see Table 2) believed that vaccinations were the primary cause of autism in their children, and this was especially true for those parents whose children showed regressive onset of ASD symptoms. These parents tended to blame heavy toxins in vaccines for their child’s condition, which is consistent with earlier studies [3]. These results also support findings reported by a recent Swedish study on perspectives of Somali mothers living in Stockholm on the MMR vaccine [93]. It was found that mothers’ decisions about whether to vaccinate their children or not were influenced by their fear about the perceived risk of developing autism, and were informed to a large extent by the opinions of relatives and friends. Goin-Kochel et al. [3] argue that until the etiology of autism is established and confirmed, the vaccine theory will likely continue to prevail among parents of children with ASD.

The analyses also revealed that parents’ perceptions of the possible etiology of their child’s ASD evolved over time–before and after diagnostic assessment for ASD, pointing to the dynamic nature of parents’ understanding of the disorder. As S1 Fig illustrates, some of the parents’ initial interpretations of ASD symptoms (e.g. colic, epileptic seizures, hearing impairment) were not mentioned after the ASD diagnosis was obtained; instead, parents attributed their children’s difficulties to other causal factors such as genetic factors or vaccinations. However, other initial interpretations remained stable over time even after a formal diagnosis was made (regardless of parents’ cultural background), e.g. the child’s reaction to parents’ separation/divorce. This finding could be explained in part by children’s developmental trajectories when behavioral signs of ASD emerge over time [94]. Knowledge about parents’ causal beliefs about ASD is important, as it allows practitioners–preschool teachers, special educators and health care professionals–to identify which of those beliefs might hinder or facilitate treatment adherence, and therefore its effectiveness.

### Parental help-seeking strategies and treatment decisions

The findings illustrate that irrespective of their region of origin, socio-economic status and perceptions of their child’s condition, all interviewed parents expressed the need to help their child as soon as possible, and turned to medical professionals to achieve this. With the exception of one mother, all parents reported contacting local Child Health Care Centers (CHCCs) to discuss their concerns with nurses, pediatricians or psychologists, who in turn referred children and their families to regional Child and Adolescent Psychiatric Clinics (CAPCs) for assessment and confirmation of ASD diagnosis. These findings support earlier research [95] suggesting that parental help seeking for children’s behavioral problems can be influenced by a country’s health care system, and could therefore vary in different cultural contexts depending on type of insurance and access to a publicly-funded formal support system.

All families in the present study reported that their children received various types of publicly-funded supports and services, such as interventions based on applied behavioral analysis (ABA), speech therapy, personal assistance, relief service, transportation services, and accommodation. This is not surprising, as children with ASD in Sweden are entitled to free support services ensured by several pieces of national legislation [96]. Research has shown that parents of children with ASD are more likely to choose government-funded interventions than self-funded [97]. Indeed, in the present study, some families from an immigrant background mentioned the high cost of available ASD interventions in their home countries. Carlon et al. [98] argue that parents’ treatment decisions can be influenced by such factors as availability and accessibility of interventions and of alternative treatments, the cost of interventions, and available funding, as well as the specific needs of the child. Parents in our study stressed the importance of speech therapy for their children’s language skills acquisition. However, inaccessibility of treatment due to long waiting lists, dissatisfaction with the quality of services, and continual rejections of applications for personal assistance for their children had led some parents to seek out private services or pursue other treatment choices, such as complementary and alternative treatments–a finding consistent with previous studies [99–101]. For instance, parents mentioned nutritional supplements and gluten- and dairy-free diets as the most frequently used complementary treatments. Some of the complementary and alternative treatments for ASD have been described in the literature as either emerging or unestablished, i.e. lacking sufficient evidence to be considered as evidence-based practices [102]. Research has indicated that there is still little evidence to support the use of dietary interventions or nutritional supplements for children with ASD [103], although these types of complementary treatment are most often chosen by parents for their children [7]. Moreover, some alternative treatments are not only ineffective, but also potentially dangerous, such as hyperbaric oxygen therapy, the use of which may lead to paralysis and air embolism [101]. In the present study, one parent reported using this treatment for her child. These findings are of importance for clinical practice: health care professionals should engage parents of children with ASD in open and respectful dialogues concerning parental beliefs about ASD etiology and decisions on the use of any non-conventional treatment strategies [9, 101]. This can prevent use of ineffective and potentially harmful treatments as alternatives to well-established, evidence-based practices.

The findings revealed that almost all families (and predominantly mothers) were actively engaged in delivering home-based interventions to their children with ASD to teach communication and social skills. This finding is in line with previous research underscoring the advantages of including parents in treatment [104]. Moreover, in Sweden the importance of involving parents in early intervention delivery is highlighted in recommendations on early intervention practices for preschool-aged children with ASD by the Association for habilitation services in Sweden [105]. Indeed, parents in this study reported using strategies learned from the habilitation center professionals with their children in home settings. Future research could explore the effectiveness of parent-mediated interventions within the Swedish context.

Many parents in the present study who described themselves as religious or spiritual reported that their faith helped them cope and gave them strength to proceed in their difficult life situations. Studies involving immigrant Muslim families with children with ASD have shown that parents’ religious faith played a crucial role in helping them to accept their child’s condition, giving meaning to their experiences and providing hope for their children “to get better” [66, 67, 106]. Similar experiences have been reported by families observing Christian religious traditions [29]. This result is important for understanding resilience factors that help parents cope with stressful situations when caring for children with ASD [107], and should therefore be taken into account by professionals when planning and providing family-centered interventions.

In summary, the overall patterns of findings of the present study suggest that the development of families’ explanatory models of autism in Sweden was shaped by a complex interplay of several sociocultural factors found in children’s and their families’ proximal and distal environments. Social interactions between parents, children, extended family members, educational and health care professionals, people in families’ broader social network, and interactions with macrosystemic environmental influences such as media and national laws or regulations may have contributed to the formation of parents’ explanations for the disorder. None of these factors in isolation can explain the differences or commonalities in explanatory models across families in this study. For instance, similar patterns were observed for *all* parents irrespective of their ethnic/cultural background in terms of time and mode of symptom onset, seeking help within the Swedish formal health care system, and early intensive educational interventions as preferred treatments for their children to achieve positive development and acquisition of skills. Instead, other factors such as parents’ education, occupation and gender may have played a larger role in formation of explanatory models for their children’s ASD, thus being either a facilitator or a barrier to help seeking. At the same time, parents’ explanatory models should also be viewed through the lens of time as a contributing factor in order to understand how these models were formed. As the results show, parents’ understanding and perception of autism evolved over time, in interaction with various sources of information about autism and parents’ previous knowledge of the disorder. The results indicate that for many parents, the diagnosis of autism became a tool that helped them explain their child’s condition to other people in their formal and informal social networks, and also a tool for seeking out appropriate treatments. One could also argue that parents’ cultural/ethnic background might have played a certain role in the formation of explanatory models *after* the child obtained an ASD diagnosis. As the results show, Swedish parents and parents from other Western European countries did not report seeking alternative explanations for their children’s autism (also reflected in self- and family prototypes) and non-established treatments, unlike some parents with a non-Western European cultural background, who reported using certain non-evidence-based practices. It is possible that parents’ negative experiences of the Swedish support system–educational, health care or social welfare–might have contributed to the development of a feeling of distrust of the system, leading some of them to look for a “second opinion” to confirm the diagnosis or to seek alternative treatments for their child’s condition. Future research is needed to investigate the relationships between cultural factors and families’ treatment choices for their children in Sweden and other Nordic countries.

### Implications for research and practice

The present study has demonstrated the validity of using the Explanatory Model supplementary module in research to further understand explanatory models of autism held by parents of young children in the cultural context of Sweden. As mentioned earlier, the results of the study confirm that explanatory models can change over time, and can be formed by various contextual influences [19]. In addition, the results show that all interviewed families were able to relate their child’s problems to prototypical experiences of others, which is in line with previous research on prototype narratives in mental illness [17]. In addition, the findings point to the existence of social barriers to obtaining timely assessment for ASD, e.g. due to stigmatization. Kirmayer [108] noted that there is a need for research on the CFI in order to refine its components. We believe that the results of the present study could be informative for future research on the use of the CFI-Informant version or the Explanatory Model supplementary module. For instance, family studies in the field of ASD research have described a genetic liability for ASD among relatives of individuals with autism who did not have a diagnosis of autism but who displayed personality, behavioral and language characteristics that were milder but qualitatively similar to the core features of the disorder–a concept known in the literature as the *broad autism phenotype* (BAP) [109–111]. In the present study, we use the terms “family prototypes” and “self-prototypical experiences”, borrowed from the McGill Illness Narrative Interview (MINI) [17], to denote parents’ own or other family members’ experiences of social and behavioral traits analogous to their children’s ASD core symptoms. Therefore, we suggest that future studies employing the CFI framework and involving families of young children with autism could use the concept of the BAP as an extension to the concept of prototypes included in the supplementary module. Furthermore, a newly, analytically-emerged subcategory labeled in this study as “typically developing children prototypes” could be used to denote the strategy used by parents to understand their children’s problem behavior of comparing them with typically developing peers or siblings.

The results of this study also point to the potential of the CFI’s components to be utilized in clinical practice: it can complement the diagnostic assessment of ASD by obtaining additional information about families’ cultural context [4]. In Sweden, leading experts in cultural formulation encourage the use of the CFI and its supplementary modules in order to increase practitioners’ cultural awareness. For instance, the Swedish translation of the CFI is available to practitioners free of charge; more importantly, information about the CFI is included in guidelines for health care [112]. This has direct implications for practitioners, including special educators, working with families of young children with ASD in health care settings such as child habilitation centers. By interacting with families and gaining trust, special educators could elicit parents’ cultural explanations of their children’s disorder to understand what treatment expectations families may have. For instance, in the present study, some mothers did not consider psychoeducational support for parents provided at habilitation centers as particularly helpful; instead, they expected more intensive sessions of speech and language therapy for their children. By listening to parents’ rationale for their preferred intervention strategies, professionals, including special educators, could facilitate a process of negotiation and shared decision-making, and thus increase treatment engagement and adherence [19].

The study findings can also inform CHCC nurses and public health professionals regarding the use of vaccinations. As our data show, some parents from an immigrant background tended to blame vaccines for their child’s ASD. It is important for CHCC nurses to build empathic rapport with culturally diverse parents of young children, as research shows that mothers’ trust in the nurses can be a crucial aspect in their decision whether or not to vaccinate their children [94]. It is essential that health care professionals’ conversations about vaccinations with parents of children suspected for ASD occur in conjunction with discussions about ASD assessment [92].

### Limitations of the study

The present study has several limitations. Firstly, the study results are not generalizable to all parents with young children diagnosed with ASD in Sweden in terms of socio-demographic characteristics, e.g. level of education: more than a half of all participants (n = 10) held a university degree. Secondly, the study’s sample size of 15 families consisting of non-representative numbers of parents with diverse cultural backgrounds (including Swedish) did not make it possible to explore intra-cultural differences in parental explanatory models of ASD that otherwise might exist due to other factors, e.g., parents’ socio-economic status or level of education. Furthermore, for the majority of families, the identified causal explanations for autism were idiosyncratic, i.e. unique to each family member. On the other hand, these individual beliefs expressed by the parents could be viewed as a strength of the study, supporting arguments for the necessity of designing individualized, culturally sensitive interventions, tailored to the unique needs of each child with ASD and his/her family members [113]. Moreover, our analyses revealed striking commonalities across all families regarding parents’ strategies for understanding children’s behavior and seeking help, irrespective of parents’ cultural and religious affiliation, socio-economic status or level of education, which we consider a strength of the study. Another limitation is that the results do not provide a comprehensive picture of parents’ treatment decisions in order to understand the extent to which parents were engaged or disengaged with interventions suggested by CHCC professionals, given that some parents from an immigrant background expressed dissatisfaction with interventions. Moreover, due to the study’s cross-sectional design, it was not possible to determine whether parents could sustain the use of certain complementary and alternative treatments such as diet interventions or homeopathic medicine over time. Despite these limitations, the study findings provide a glimpse into parents’ treatment choices based on their perceptions of causal beliefs about autism. By using rigorous longitudinal, cross-cultural research designs and involving more families representing the cultural heterogeneity of the current population in Sweden, future studies could tap better possible inter- and intracultural differences and commonalities in parental explanatory models. This in turn could help professionals to understand further the impact of cultural factors on assessment and interventions for children with ASD.

## Supporting information

S1 Table Questions to elicit patients’ explanatory models of illness as proposed by Kleinman [13]. (DOCX)

S2 Table Explanatory Model Supplementary module 1 in DSM-5. Adapted from Lewis-Fernández et al. [21].
(DOCX)

S3 Table Questions to elicit parents’ explanatory models of autism. Adapted from Levy et al. [23].
(DOCX)

S1 Fig Parents’ perceived causal explanations to their children’s condition before and after diagnosis of ASD. (DOC)
