# Supplementary material for: Culturally diverse families of young children with ASD in Sweden: Parental explanatory models
Source: PLoS One. 2020 Jul 27;15(7):e0236329. doi: 10.1371/journal.pone.0236329 (PMC7384670; doi:10.1371/journal.pone.0236329)
Supplement: S1 Table — (DOCX) [file pone.0236329.s001.docx]

**S1 Table .** Questions to elicit patient’s explanatory models of illness as proposed by Kleinman [13].

| 1. | What do you think has caused your problem? |
| --- | --- |
| 2. | Why do you think it started when it did? |
| 3. | What do you think your sickness does to you? How does it work? |
| 4. | How severe is your sickness? Will it have a short or long course? |
| 5. | What kind of treatment do you think you should receive? |
| 6. | What are the most important results you hope to receive from this treatment? |
| 7. | What are the chief problems your sickness has caused for you? |
| 8. | What do you fear most about your sickness? |
