# Supplementary material for: Culturally diverse families of young children with ASD in Sweden: Parental explanatory models
Source: PLoS One. 2020 Jul 27;15(7):e0236329. doi: 10.1371/journal.pone.0236329 (PMC7384670; doi:10.1371/journal.pone.0236329)
Supplement: S2 Table — Adapted from Lewis-Fernández et al. [21]. (DOCX) [file pone.0236329.s002.docx]

**S2 Table .** Explanatory Model Supplementary module 1 in DSM-5. Adapted from Lewis-Fernández et al.[21]

| 1. **Explanatory Model** |
| --- |
| ***GUIDE TO INTERVIEWER:*** *This module aims to clarify the individual’s understanding of the problem based on his or her ideas about cause and mechanism (explanatory models) and past experiences of, or knowing someone with, a similar problem (illness prototypes). The individual may identify the problem as a symptom, a specific term, or expression (e.g. “nerves”, “being on edge”), a situation “e.g. loss of a job), or a relationship (e.g. conflict with others). In the examples below, the individual’s own words should be used to replace “[PROBLEM]”. If there are multiple problems, each relevant problem can be explored. The following questions may be used to elicit the individual’s understanding and experience of that problem or predicament.* |
| **INTRODUCTION FOR THE INDIVIDUAL BEING INTERVIEWED:** I would like to understand the problems that bring you here so that I can help you more effectively. I will be asking you some questions to learn more about your own ideas about the causes of your problems and they way they affect your daily life. |
| ***General understanding of the problem***   1. Can you tell me about how you understand your [PROBLEM]? 2. What did you know about your [PROBLEM] before it affected you? |
| ***Illness prototypes***   1. Have you ever had anything like your [PROBLEM] before? Please tell me about it. 2. Do you know anyone else, or heard of anyone else, with this [PROBLEM]? If so, please describe that person’s [PROBLEM] and how it affected that person. Do you think this will happen to you too? 3. Have you seen on television, heard on the radio, read in a magazine, or found on the internet anything about [PROBLEM]. Please tell me about it. |
| ***Causal explanations***   1. Can you tell me what you think caused your [PROBLEM]? (*PROBE AS NEDDED*: Is there more than one cause that may explain it?) 2. Have your ideas about the cause of the [PROBLEM] changed? How? What changed your ideas about the cause? 3. What do people in your family, friends, or others in your community think caused the [PROBLEM]? (*PROBE AS NEDDED*: Are there ideas about it different from yours? How so?) 4. How do you think your [PROBLEM] affects your body? Your mind? Your spiritual well-being? |
| ***Course of illness***   1. What usually happens to people who have this [PROBLEM]? In your own case, what do you think is likely to happen? 2. Do you consider your [PROBLEM] to be serious? Why? What is the worst that could happen? 3. How concerned are other people in your family, friends or community about your having this [PROBLEM]? Please tell me about that. |
| ***Help seeking and treatment expectations***   1. What do you think is the best way to deal with this kind of problem? 2. What do your family, friends or others in your community think is the best way of dealing with this kind of problem? |
