# Supplementary material for: Culturally diverse families of young children with ASD in Sweden: Parental explanatory models
Source: PLoS One. 2020 Jul 27;15(7):e0236329. doi: 10.1371/journal.pone.0236329 (PMC7384670; doi:10.1371/journal.pone.0236329)
Supplement: S3 Table — Adapted from Levy et al. [23]. (DOCX) [file pone.0236329.s003.docx]

**S3 Table .** Questions for understanding parents’ beliefs about autism. Adapted from Levy et al. [23].

| 1. | What do you call your child’s problem before it was diagnosed? |
| --- | --- |
| 2. | What do you think caused it? |
| 3. | Why do you think it started when it did? |
| 4. | What do you think autism does? How does it work? |
| 5. | How severe is it? Will it have a short or long course? |
| 6. | What are the chief problems your child’s autism has caused? |
| 7. | What do you fear most about it? |
| 8. | What kind of treatment do you think your child should receive? What do you expect from this treatment? |
