# Supplementary material for: Culturally diverse families of young children with ASD in Sweden: Parental explanatory models
Source: PLoS One. 2020 Jul 27;15(7):e0236329. doi: 10.1371/journal.pone.0236329 (PMC7384670; doi:10.1371/journal.pone.0236329)
Supplement: S1 Fig — (DOC) [file pone.0236329.s004.doc]

**S1 Figure** *.* Parents’ perceived causal explanations to their children’s condition before and after diagnosis of ASD.

| Problem definitions ***before*** diagnostic assessment |  |  | Causes of ASD ***after*** diagnosis obtained |  | “I don’t know”/”I cannot say for sure” |  | **Unknown** |
| --- | --- | --- | --- | --- | --- | --- | --- |
|  | **Child’s condition:**   - epileptic seizures - baby colic - hearing impairment - insomnia   **Reaction to external environmental influences:**   - stem-cells transplantation; - exposure to several languages; - physical abuse by a peer at   preschool;   - measles infection during trip to   mother’s home country   - Reaction to parents’ separation or divorce |  |  |  | Genetic/Hereditary  Vaccinations  Supernatural/religious  Medication overdose |  | **Definite Causes** |
|  |  |  |  |  | Birth complications  Congenital damage (at prenatal stage)  Reaction to gluten  Reaction to genetically modified food  Vitamin D deficiency  Reaction to parents’ separation  Reaction to inadequate educational support (causing ID)  Reaction to physical abuse by peer at preschool  Head trauma (“fell down when was a baby”) |  | **Possible causes** |
|  | **Lack of knowledge on parental skills**  **Cultural differences in childcare** |  |  |  |  |  |  |
|  |  |  |  |  |  |  |  |
|  |  |  |  |  |  |  |  |
